# Disruption of TLR3 Signaling Due to Cleavage of TRIF by the Hepatitis A Virus Protease-Polymerase Processing Intermediate, 3CD

**DOI:** 10.1371/journal.ppat.1002169

**Published:** 2011-09-08

**Authors:** Lin Qu, Zongdi Feng, Daisuke Yamane, Yuqiong Liang, Robert E. Lanford, Kui Li, Stanley M. Lemon

**Affiliations:** 1 Lineberger Comprehensive Cancer Center and the Division of Infectious Diseases, Department of Medicine, The University of North Carolina at Chapel Hill, Chapel Hill, North Carolina, United States of America; 2 Department of Virology and Immunology, Texas Biomedical Research Institute, San Antonio, Texas, United States of America; 3 Department of Microbiology, Immunology and Biochemistry, University of Tennessee Health Science Center, Memphis, Tennessee, United States of America; Columbia University, United States of America

## Abstract

Toll-like receptor 3 (TLR3) and cytosolic RIG-I-like helicases (RIG-I and MDA5) sense viral RNAs and activate innate immune signaling pathways that induce expression of interferon (IFN) through specific adaptor proteins, TIR domain-containing adaptor inducing interferon-β (TRIF), and mitochondrial antiviral signaling protein (MAVS), respectively. Previously, we demonstrated that hepatitis A virus (HAV), a unique hepatotropic human picornavirus, disrupts RIG-I/MDA5 signaling by targeting MAVS for cleavage by 3ABC, a precursor of the sole HAV protease, 3C^pro^, that is derived by auto-processing of the P3 (3ABCD) segment of the viral polyprotein. Here, we show that HAV also disrupts TLR3 signaling, inhibiting poly(I:C)-stimulated dimerization of IFN regulatory factor 3 (IRF-3), IRF-3 translocation to the nucleus, and IFN-β promoter activation, by targeting TRIF for degradation by a distinct 3ABCD processing intermediate, the 3CD protease-polymerase precursor. TRIF is proteolytically cleaved by 3CD, but not by the mature 3C^pro^ protease or the 3ABC precursor that degrades MAVS. 3CD-mediated degradation of TRIF depends on both the cysteine protease activity of 3C^pro^ and downstream 3D^pol^ sequence, but not 3D^pol^ polymerase activity. Cleavage occurs at two non-canonical 3C^pro^ recognition sequences in TRIF, and involves a hierarchical process in which primary cleavage at Gln-554 is a prerequisite for scission at Gln-190. The results of mutational studies indicate that 3D^pol^ sequence modulates the substrate specificity of the upstream 3C^pro^ protease when fused to it *in cis* in 3CD, allowing 3CD to target cleavage sites not normally recognized by 3C^pro^. HAV thus disrupts both RIG-I/MDA5 and TLR3 signaling pathways through cleavage of essential adaptor proteins by two distinct protease precursors derived from the common 3ABCD polyprotein processing intermediate.

## Author Summary

While viruses that target the liver often cause lengthy infections with considerable morbidity, there is limited understanding of how they evade host responses. We have studied hepatitis A virus (HAV), an important cause of acute hepatitis in humans. Although HAV infection typically results in hepatic inflammation, there is no disease in the liver during the first weeks of infection despite robust virus replication. This suggests that HAV either fails to stimulate or efficiently evades recognition by host innate immune sensors. Our prior work showed HAV disrupts RIG-I/MDA5 signaling by targeting MAVS, an essential adaptor protein, for degradation by 3ABC, a precursor of the only HAV protease, 3C^pro^. Here, we show here that a distinct viral processing intermediate, the 3CD protease-polymerase, disrupts TLR3 signaling by degrading its adaptor protein, TRIF. HAV has evolved a novel strategy to target two different host adaptor proteins with a single protease, using its 3D^pol^ RNA polymerase to modify the substrate specificity of its 3C^pro^ protease when fused to it in the 3CD precursor, thus allowing it to target non-canonical 3C^pro^ recognition sequences in TRIF. This remarkable example of viral adaptation allows the virus to target two different host adaptor proteins with a single viral protease.

## Introduction

Hepatitis A virus (HAV) [1] and hepatitis C virus (HCV) [2] are positive-strand RNA viruses that cause hepatitis in humans. Despite important differences in virion structure, they share similar genome structures and many aspects of their replication strategies. Both viruses demonstrate strong tropism for the hepatocyte, and replicate their RNA genomes in replicase complexes contained within cytoplasmic vesicles. Both produce double-stranded RNA (dsRNA), a potent pathogen-associated molecular pattern (PAMP) recognized by innate immune sensors, as replication intermediates. Thus, both HAV and HCV face similar challenges posed by the innate immune system early in the course of hepatic infection. However, HAV and HCV infections have dramatically different outcomes. HAV never causes chronic hepatitis while HCV does so in the majority of those it infects. Prolonged shedding of HAV has been reported in premature infants [3], but long-term persistent infection has never been documented. This contrasts sharply with HCV, which persists for decades in the majority of those infected [2], [4].

Although factors controlling HCV infection outcome are poorly understood, T cell responses are critical [reviewed in 2]. T cells also appear to be important for HAV clearance [5], [6]. In both cases, the vigor and breadth of the virus-specific T response is likely to be profoundly influenced by early interferon (IFN) and other cytokine responses evoked by innate antiviral response pathways. How HCV both induces and disrupts signaling initiated by retinoic acid-inducible gene I (RIG-I) and Toll-like receptor 3 (TLR3) has been studied in depth. Proteolytic cleavage of mitochondrial antiviral signaling protein (MAVS, also known as IPS-1, VISA or Cardif) and TIR domain-containing adaptor inducing IFN-β (TRIF, also known as TICAM-1) by the NS3/4A serine protease of HCV effectively blocks the activation of IFN-regulatory factor 3 (IRF-3) and nuclear factor-κB (NF-κB) induced by RIG-I and TLR3, respectively [7], [8], [9]. Much less is known about how HAV stimulates or antagonizes these innate signaling pathways.

Both clinical and experimental observations indicate that there is extensive replication of HAV within the liver prior to the onset of hepatic inflammation 3–4 weeks after infection [10]. This lengthy, clinically silent incubation period suggests that HAV either blocks or otherwise fails to induce innate immune responses to dsRNA in the early stages of the infection. Consistent with this, HAV, like HCV, disrupts virus-induced signaling initiated by RIG-I-like receptors (RLRs, RIG-I and melanoma differentiation associated gene 5, MDA-5) [11], [12]. Our previous work shows that it does this by targeting MAVS for proteolysis by a precursor of its 3C^pro^ cysteine protease, 3ABC [12]. Cleavage requires both the protease activity of 3C^pro^ and a transmembrane domain in 3A that directs 3ABC to the mitochondrial outer membrane where MAVS is localized [12]. The shared capacity of the HAV 3ABC and HCV NS3/4A proteases to cleave MAVS and disrupt signaling from RLRs suggests that MAVS-dependent signaling is critical to antiviral defense in the liver (as it is in other tissues), but also indicates that NS3/4A cleavage of MAVS is not primarily responsible for the unique ability of HCV to establish persistent infections.

In addition to RLRs, TLR3 is functionally expressed in primary human hepatocytes [13]. During HCV infection, signaling initiated by TLR3 recognition of dsRNA is blocked by NS3/4A cleavage of the adaptor protein, TRIF [7], [13]. This led us to ask whether HAV also antagonizes TLR3 signaling. We show here that HAV strongly inhibits TLR3 signaling by also targeting TRIF for degradation. We demonstrate that TRIF is proteolytically cleaved by a distinct intermediate in the polyprotein processing cascade, the viral 3CD protease-polymerase. Cleavage requires expression of the cysteine protease activity of 3C^pro^ fused *in cis* to 3D^pol^ sequence. Mutational studies reveal an unexpected role of the 3D^pol^ domain in modulating the substrate specificity of 3C^pro^ such that it is able to achieve scission of non-canonical 3C^pro^ cleavage sites within TRIF. The role played by the polymerase sequence in innate immune evasion represents a remarkable and unique mechanism of viral adaptation to the intrahepatic environment, and provides a second major evasive strategy by which HAV can escape innate immunity.

## Results

### HAV inhibits TLR3 signaling by reducing abundance of the adaptor protein TRIF

**Figure 1:**
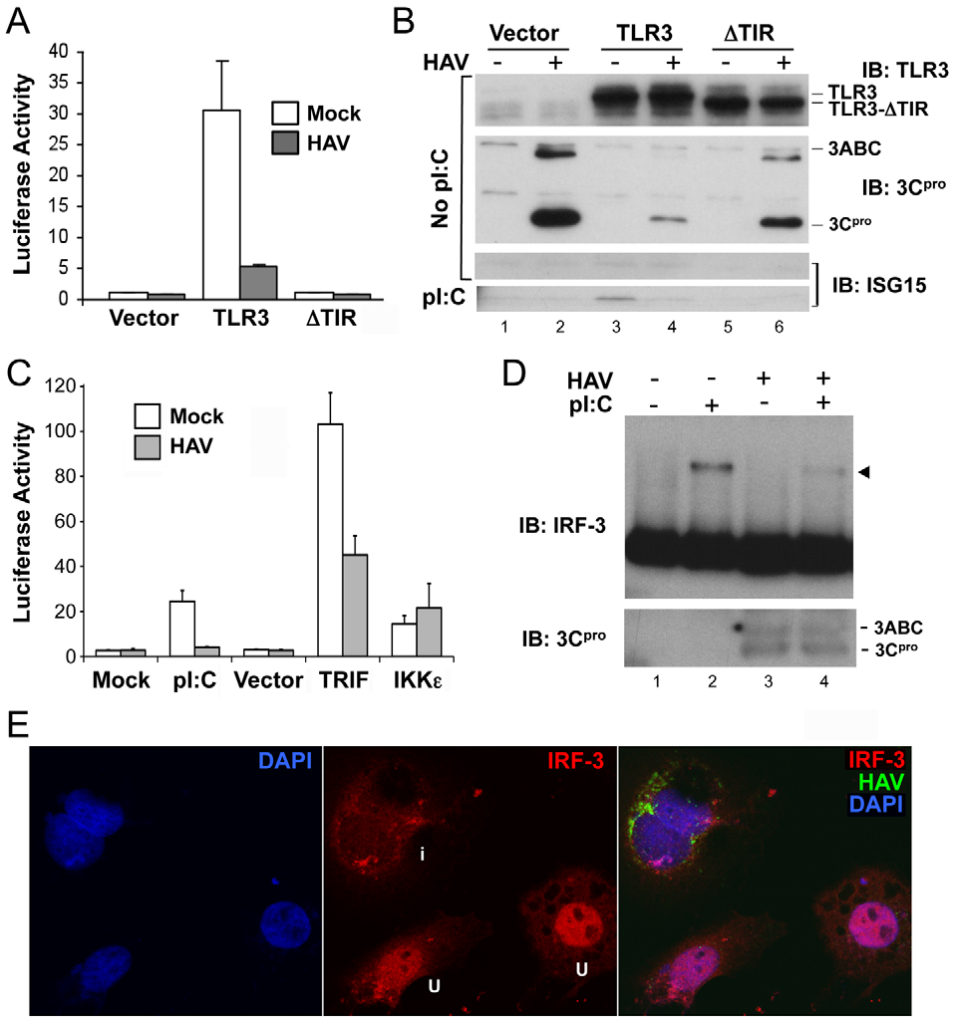
HAV inhibits TLR3 signaling prior to IRF-3 phosphorylation and nuclear translocation. (**A**) IFN-β-Luc reporter assay of Huh7-TLR3, Huh7-ΔTIR, and Huh7-Vector cells that were mock- or HAV-infected (m.o.i. = 3) for 5 days and stimulated with extracellular poly(I:C) for 6 hours. Luciferase activity was normalized to an internal β-gal transfection control and is presented fold-induction by poly(I:C). (**B**) Immunoblots of the cells in panel (A) showing expression of TLR3 and TLR3-ΔTIR (top panel), HAV 3ABC and 3C^pro^ (middle panel), and poly(I:C)-induced ISG15 (bottom panel). (**C**) IFN-β-Luc reporter assay of poly(I:C), TRIF, or IKKε-induced activation of the IFN-β promoter in mock- or HAV-infected Huh7-TLR3 cells. (**D**) HAV infection blocks poly-(I:C)-induced dimerization of IRF-3 in Huh7/TLR3 cells. (top panel) Immunoblot of IRF-3 in extracts of HAV- or mock-infected cells resolved by native PAGE. Cells were lysed 2 hrs after addition of poly-(I:C) (50 µg/ml) to media. IRF-3 dimers are indicated by the arrowhead. (bottom panel) Immunoblot of native PAGE with 3C^pro^-specific antibody. (**E**) Laser-scanning confocal microscopy images of Huh7-TLR3 cells infected with HAV at low m.o.i. for 5 days and stimulated with poly-(I:C). Cells were labeled with antibodies to IRF-3 (red) and HAV (green), while nuclei were counterstained with DAPI (blue). The merged image on the right shows that IRF-3 has undergone nuclear translocation in two uninfected (‘u’, see middle panel) cells, but not in an infected cell (‘i’) containing HAV antigen.

Although hepatocytes express TLR3, Huh7 hepatoma cells, which are permissive for replication of cell culture-adapted HAV, are defective in TLR3 signaling [14], [15]. We therefore studied the impact of HAV infection on TLR3 signaling in Huh7 cells in which signaling was functionally reconstituted by retroviral transduction of TLR3 expression [13]. Previous studies of these cells include extensive control experiments showing that the activation of IRF-3 by extracellular poly-(I:C) occurs specifically through TLR3 signaling [13]. Control cells used in here included Huh7 cells transduced in parallel with a TIR-domain TLR3 deletion mutant or empty vector. Stimulation of Huh7-TLR3 cells with extracellular poly(I:C), a synthetic dsRNA analog, induced transcriptional activation of the IFN-β promoter and expression of the interferon-stimulated gene (ISG) ISG15, neither of which were observed in Huh7-ΔTIR or Huh7-Vector cells (Fig. 1A, B). However, prior infection of Huh7-TLR3 cells with HM175/18f, a cell culture-adapted HAV variant [16], strongly inhibited both responses (Fig. 1A, B). The expression levels of TLR3 and its ΔTIR mutant were not affected by HAV (Fig. 1B), suggesting that HAV does not disrupt TLR3 signaling by reducing TLR3 abundance. HAV inhibition of TLR3 signaling was also observed in Huh7.5-TLR3 cells, TLR3-reconstituted Huh-7.5 cells that are deficient in RIG-I signaling (Fig. S1A in Text S1) [13].

The IFN-β promoter is activated by overexpression of the TLR3 adaptor protein, TRIF, or downstream kinases, TBK-1 or IKKε, that phosphorylate IRF-3 [17]. In HAV-infected cells, however, its activation by TRIF was reduced by about 50%, while there was no reduction in its stimulation by IKKε (Fig. 1C). This result is similar to that reported previously by Fensterl et al. [11]. We also observed a marked reduction in the abundance of IRF-3 dimers in HAV-infected Huh7-TLR3 cells stimulated with extracellular poly-(I:C) (Fig. 1D). In addition, confocal microscopy revealed that IRF-3 did not undergo nuclear translocation upon poly-(I:C) stimulation of HAV-infected Huh7-TLR3 cells (Fig. 1E), while this occurred uniformly in uninfected cells (Fig. S1B in Text S1, right panel). Importantly, HAV infection itself induced neither IRF-3 dimerization (Fig. 1D) nor nuclear translocation (Fig. S1B in Text S1, left panel), indicating an absence of IRF-3 activation. Collectively, these results indicate that HAV infection disrupts the signal transduction pathway from TLR3 prior to the kinases responsible for IRF-3 activation.

**Figure 2:**
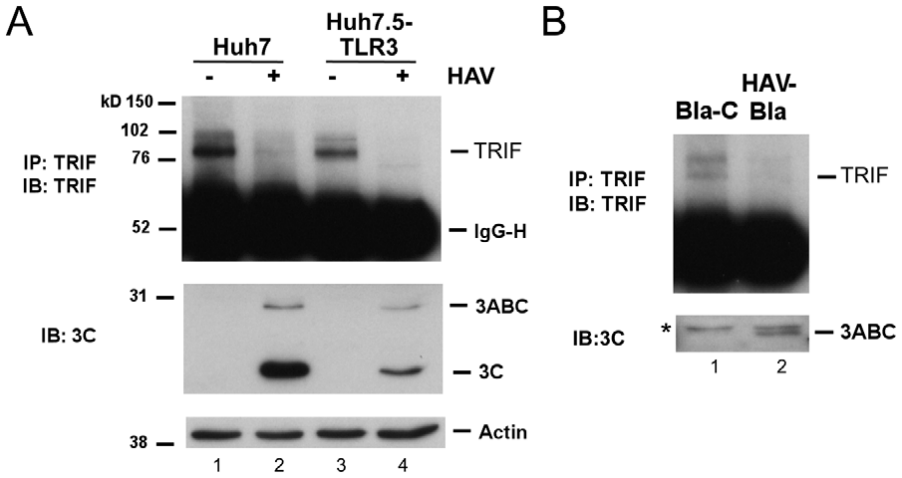
TRIF abundance is reduced by HAV infection. (**A**) (top panel) Endogenous TRIF in mock- or HAV-infected Huh7 and Huh7.5-TLR3 cells was immunoprecipitated using a rabbit anti-TRIF antibody [7] and detected by immunoblotting. (middle panel) HAV infection was confirmed by immunoblot of viral proteins 3ABC and 3C^pro^. (bottom panel) Actin served as loading control. (**B**) Endogenous TRIF and 3ABC expression in the HAV replicon HAV-Bla cells and IFN-α-cured Bla-C cells [12] (top). TRIF was detected as in panel A. ‘*’ indicates a nonspecific protein band.

Consistent with a defect in signaling at this level, we found the abundance of endogenous TRIF was substantially reduced in HAV-infected Huh7 or Huh7.5-TLR3 cells (Fig. 2A). In addition, we could not detect TRIF in a stable Huh7 cell line harboring an autonomously replicating subgenomic HAV replicon (HAV-Bla cells) [18], while TRIF expression was restored after eliminating the replicon by IFN treatment (Bla-C cells, Fig. 2B). Thus, HAV infection disrupts TLR3 signaling by substantially decreasing the expression of TRIF.

### The HAV 3CD protease-polymerase processing intermediate disrupts TLR3 signaling by cleaving TRIF

**Figure 3:**
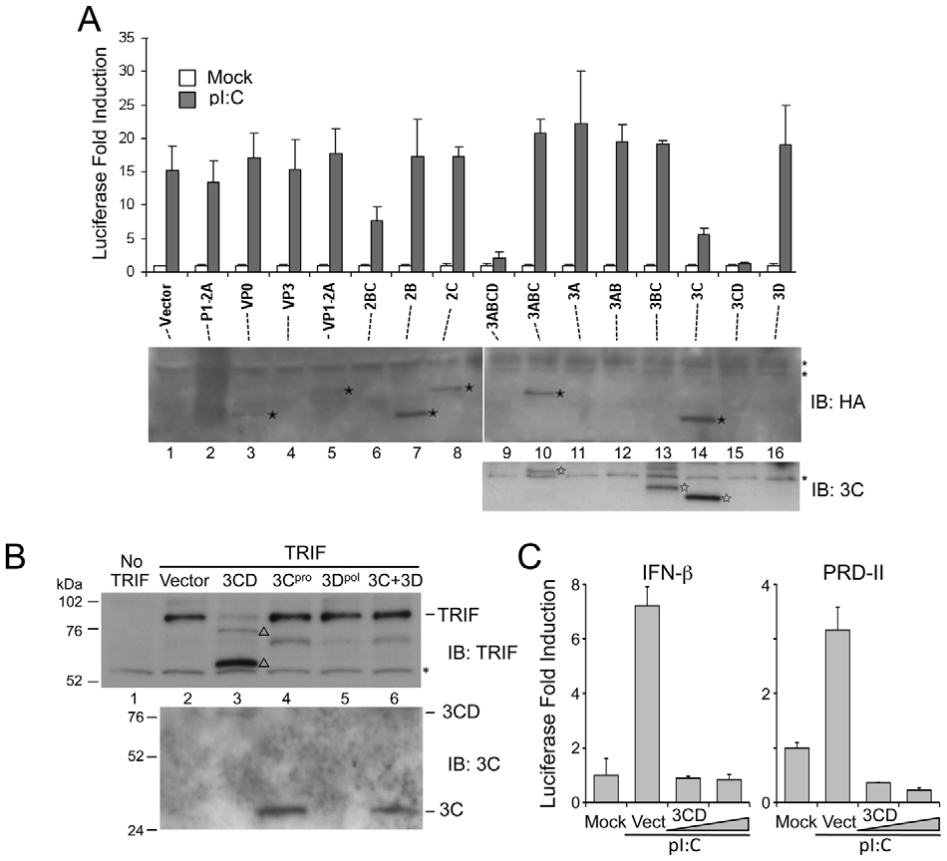
The HAV 3CD protease-polymerase precursor disrupts TLR3 signaling through cleavage of TRIF. (**A**) (top panel) IFN-β-Luc reporter assay of Huh7.5-TLR3 cells transfected with expression vectors encoding HA-tagged HAV proteins and stimulated with extracellular poly(I:C). (bottom panel) Extracts of similarly transfected cells were analyzed by immunoblotting with HA antibody to detect HAV proteins (solid star, middle panel), or with specific antibody to 3C^pro^ (open star, bottom panel). ‘*’ indicates a nonspecific protein band. 3CD and 3D^pol^ were not visualized with any antibody. (**B**) HEK 293FT cells were co-transfected with vectors expressing TRIF and HA-tagged HAV proteins. Cell lysates were analyzed by immunoblotting for TRIF (top) or 3C^pro^ and 3CD (bottom). In addition to the 90-kDa full-length TRIF, two TRIF fragments, 75- and 55-kDa in size (‘Δ’), were detected in cells expressing 3CD. A nonspecific protein band detected by TRIF antibody (‘*’) indicates equal loading. (**C**) IFN-β-Luc and PRD-II-Luc (NF-κB specific) reporter assays of Hela cells transfected with control or 3CD expression vectors and stimulated with extracellular poly(I:C). Increasing amounts of 3CD expression vector (50 ng and 100 ng) were used.

To determine whether a specific HAV protein or polyprotein processing intermediate was responsible for the reduction in TRIF abundance and, as a result, the inhibition of TLR3 signaling, we over-expressed individual proteins in Huh7-TLR3 cells, assessing the impact on poly(I:C)-induced, TLR3-dependent activation of the IFN-β promoter. While ectopic expression of the P1-2A structural proteins (P1-2A, VP0, VP3, VP1-2A) and 2B or 2C had little impact on TLR3 signaling, the processing intermediate 3ABCD and its 3CD protease-polymerase derivative strongly blocked promoter activation (Fig. 3A, top panel). An intermediate degree of suppression was observed with 2BC and 3C^pro^ expression. The effect of 2BC on signaling may be related to its capacity to induce intracellular membrane rearrangement [19], and was not studied further.

The common presence of the 3C^pro^ cysteine protease domain in 3ABCD, 3CD and 3C^pro^ suggested it may play a role in disrupting TLR3 signaling. However, 3C^pro^ alone was significantly less inhibitory than either 3CD or 3ABCD (Fig. 3A, top panel, p<0.002 by Student's t test), despite being expressed in much greater abundance (Fig. 3A, lower panel). Since 3ABCD is the precursor of 3CD, its inhibitory effect on TLR3 signaling could be due entirely to 3CD. 3ABC, which is also derived from 3ABCD and targets MAVS for cleavage [12], had no effect. Consistent with these results, the co-expression of 3CD, but not 3C^pro^ or 3D^pol^, or a combination of these two proteins, resulted in a marked reduction of ectopically expressed TRIF in HEK 293FT cells (in which endogenous TRIF expression is negligible) (Fig. 3B). The reduced abundance of full-length TRIF in cells expressing 3CD was accompanied by the appearance of two TRIF fragments with apparent molecular masses of 75 and 55 kDa that were detected by an antibody recognizing residues surrounding Ser-219 (Fig. 3B, open triangles). Since full-length TRIF has a mass of ∼90-kDa, these are likely to be overlapping degradation products. An antibody to residues 4–31 of TRIF identified an additional fragment with an apparent mass of 20 kDa, likely derived from the N-terminus of TRIF (not shown). The accumulation of at least three different fragments suggests that 3CD causes multiple scission events within TRIF. 3CD-mediated cleavage of TRIF was not dependent upon the cell culture-adaptive mutations present in the 3CD sequence of the HM175/18f virus [16] used in these studies, as it was also observed with ectopically expressed, wild-type 3CD (Fig. S3 in Text S1). Consistent with these observations, 3CD overexpression resulted in a marked reduction in poly-I:C-stimulated IFN-β and PRD-II (NF-κB-responsive) promoter activity in HeLa cells (Fig. 3C). Thus, 3CD effectively antagonizes an endogenous TLR3 pathway, as well as the reconstituted pathway in Huh7-TLR3 cells.

**Figure 4:**
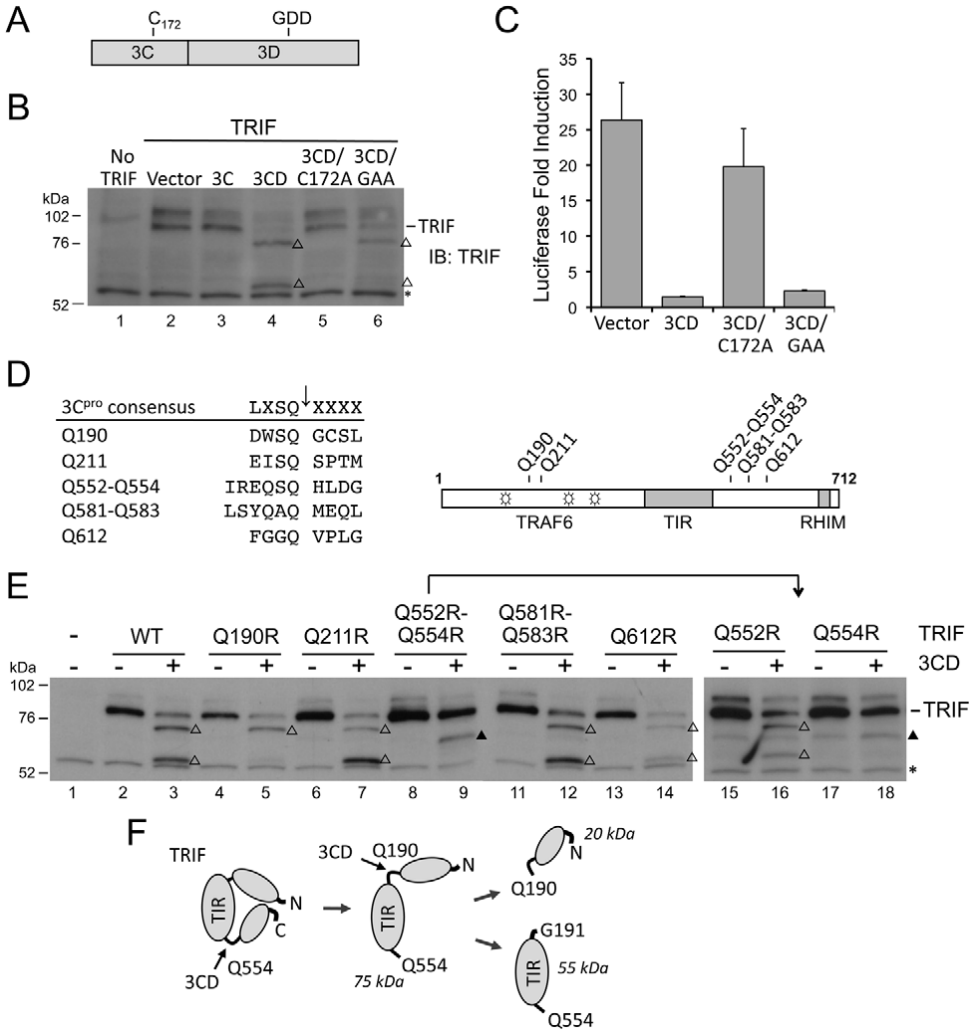
3CD cleavage of TRIF requires both 3C^pro^ protease activity and 3D^pol^ *in cis*. (**A**) Organization of 3CD, showing the position of Cys-172 at the 3C^pro^ active site and the GDD polymerase motif in 3D^pol^. (**B**) Huh7 cells were co-transfected with vectors expressing TRIF and 3C, 3D, or 3CD, as indicated. Cell extracts were analyzed by immunoblotting for TRIF. TRIF cleavage fragments are marked (‘Δ’). A nonspecific protein band detected by TRIF antibody (‘*’) indicates equal loading. (**C**) IFN-β-Luc reporter assay of Huh7-TLR3 cells transfected with vectors expressing 3CD or the related C172A and GAA mutants, and stimulated with extracellular poly-(I:C). (**D**) Alignment of 3C^pro^ consensus cleavage sequence and possible cleavage sites in TRIF (left), and locations of these sites relative to the TRAF6-binding motifs (open star) and TIR and RHIM domains (grey boxes) within TRIF (right). (**E**) Identification of Q190 and Q554 as 3CD cleavage sites in TRIF. HEK 293FT cells were co-transfected with vectors expressing TRIF mutants and 3CD, and cell extracts were analyzed by immunoblotting of TRIF. TRIF cleavage fragments and a nonspecific protein band were marked as in (B). A TRIF degradation band (solid triangle), independent of 3CD cleavage, was occasionally observed. (**F**) A model for 3CD cleavage of TRIF. Cleavage at the preferred primary Q554 site induces a conformational change that exposes the Q190 site for the second cleavage.

Since co-expression of 3C^pro^ and 3D^pol^ did not result in detectable TRIF cleavage (Fig. 3B), efficient scission appears to require expression of the protease and polymerase domains *in cis*. 3CD is known to be a catalytically active precursor of 3C^pro^ [20], thus 3CD could directly cleave TRIF. To test this hypothesis, we expressed a 3CD mutant with an Ala substitution of the active-site nucleophile, Cys-172 (Fig. 4A). This lacked any capacity to cleave ectopically expressed TRIF (Fig. 4B), confirming that the protease activity of 3CD is responsible. In contrast, a mutant in which the conserved GDD motif required for RNA-dependent RNA polymerase activity was ablated (3CD-GAA) remained capable of cleaving TRIF. The 3CD-GAA mutant also inhibited poly(I:C)-induced activation of the IFN-β promoter in Huh7-TLR3 cells, whereas the proteinase-deficient 3CD-C172A mutant did not (Fig. 4C). Collectively, the results shown in Figs. 3 and 4 indicate that TRIF cleavage results from the 3C^pro^ protease acting *in cis* with 3D^pol^.

To assess whether TRIF is cleaved by a 3C^pro^–3D^pol^ complex forming after auto-processing of 3CD, we constructed 3CD mutants with modifications at the 3C–3D junction that accelerate or retard autoprocessing. The 3C–3D junction is comprised of a primary 3C^pro^ cleavage site, IESQ^↓^R, and an alternative cleavage site, EFTQ^↓^C, separated by 9 residues (Fig. S2A in Text S1). In one mutant, 3CD-QQRR, both cleavage sites were abolished by Gln-to-Arg mutations such that expression resulted only in the 3CD precursor (Fig. S2B in Text S1). In the other, 3CD-LWG, the primary cleavage site was optimized to LWSQ↓G, making it identical to the efficiently cleaved 2C–3A junction (Fig. S2A in Text S1). Expression of 3CD-LWG led to less 3CD precursor, and a greater abundance of mature 3C^pro^ due to enhanced 3C/3D processing (Fig. S2B in Text S1). While the 3CD-QQRR mutant remained capable of cleaving TRIF, the hyper-processing 3CD-LWG mutant did not (Fig. S2C in Text S1). We conclude that the cleavage of TRIF results from the cysteine protease activity of the unprocessed 3CD sequence.

TRIF is cleaved by cellular caspases at residues D281 and D289 under conditions favoring apoptosis, including over-expression of TRIF [21]. This generates a 38-kDa fragment that is distinct from those generated by 3CD (Fig. S4A in Text S1). Moreover, a D281E-D289E (DDEE) TRIF mutant resistant to caspase cleavage [21] remained subject to cleavage by 3CD (Fig. S4A in Text S1). Thus, TRIF is not degraded indirectly by cellular caspases when 3CD is expressed. While a broadly active caspase inhibitor, z-VAD-fmk, partially inhibited the 3CD-mediated cleavage of TRIF (Fig. S4B in Text S1), z-VAD-fmk is known to inhibit cellular proteases other than caspases that, like 3C^pro^, contain cysteine nucleophiles [22].

To show that TRIF is cleaved directly by the viral protease, we attempted to purify GST-3CD and GST-3C^pro^ fusion proteins produced in *E. coli.* The GST-3CD fusion product formed an insoluble pellet upon extraction, precluding its purification. This likely reflects the extreme insolubility of 3D^pol^, which has hindered previous efforts to purify the HAV polymerase [23]. We were able to produce purified GST-3C^pro^. In cell-free cleavage assays, this demonstrated a limited ability to process [^35^S]-labeled TRIF prepared by *in vitro* translation (Fig. S5 in Text S1), but did produce the expected ∼75-, ∼55-, ∼27-, and ∼18-kDa cleavage products when incubated with full-length TRIF, or fragments representing amino acids 1–372 or 373–712 of TRIF, *in vitro* (Fig. S5B in Text S1). Taken collectively with the data shown in Fig. S4 in Text S1, these results confirm that TRIF cleavage is caused by the HAV protease directly, and not by indirect activation of a caspase or other cellular protease. The incomplete proteolysis of TRIF observed in the cell-free cleavage reactions is consistent with the partial inhibition of IFN-β promoter activation by poly-(I:C) we observed following high-level expression of 3C^pro^ in Fig. 3A. Thus, while 3C^pro^ is capable of cleaving TRIF, its capacity to do so is much less than 3CD.

### 3CD cleaves TRIF at Gln-190 and Gln-554 in an ordered process

We next examined the sequence of human TRIF for potential 3C^pro^ cleavage sites. Previous studies of 3C^pro^ substrate specificity have documented a preference for Gln at the P1 position and a consensus sequence (L,V,I)X(S,T)Q↓X where X is any amino acid [24]. The 3ABC cleavage site in MAVS, LASQ↓V, fits this consensus perfectly [12]. In contrast, TRIF does not contain any consensus 3C^pro^ cleavage sites, although several sites are partial fits that could serve as non-canonical cleavage sites with consensus P1 and P2 resides. We focused on two clusters of such sites (Fig. 4D) that could potentially generate cleavage fragments of appropriate size (75, 55, and 20 kDa, see above). We constructed a series of mutants in which the invariant Gln at each potential P1 position was substituted with Arg, and examined their cleavage by 3CD. 3CD cleavage was not affected by Q211R, Q581R-Q583R, or Q612R mutations (Fig. 4E, left), excluding these as 3CD cleavage sites. In contrast, a Q190R mutation blocked the cleavage event that generates the 55 kDa but not the 75 kDa fragment, while Q552R-Q554R mutations completely abolished 3CD cleavage of TRIF (Fig. 4E, left). We then constructed individual Q552R and Q554R mutants, and showed the loss of cleavage in Q552R-Q554R was due to Q554R (Fig. 4E, right). These results establish Q190 and Q554 as 3CD cleavage sites within TRIF, and clarify the identities of the observed TRIF cleavage fragments. The 75 kDa fragment results from cleavage at Q554 and corresponds to aa 1–554 of TRIF. This fragment is further cleaved at Q190, giving rise to the 55-kDa and 20-kDa fragments that correspond to aa 191–554 and 1–190, respectively.

The different effects of the Q190R and Q554R mutations on 3CD cleavage of TRIF indicate that 3CD cleaves TRIF in an ordered process. The fact that cleavage at Q190 cannot proceed when cleavage at Q554 is blocked (Fig. 4E) suggests that the cleavage at Q554 is a prerequisite to cleavage at Q190. We thus propose a “two-step” model for the 3CD cleavage of TRIF, in which primary cleavage at Q554 site induces a conformational change that exposes the Q190 site, allowing the second cleavage to occur (Fig. 4F).

The N-terminal region of TRIF contains three TRAF6-binding motifs that are important for activation of the transcription factors NF-κB and IRF-3 in TLR3 signaling [25], [26]. Q190 is located between the first and second of these TRAF6-binding motifs, while the Q554 cleavage site is located between the TIR domain and RHIM motif (Fig. 4D, right), both important for transcriptional activation of IFN-β [27]. Cleavage at these residues could yield fragments with reduced signaling ability, or potentially dominant negative activity against signal transduction. We thus ectopically expressed the predicted, individual 3CD-generated TRIF fragments: N-190 (aa 1–190), N-554 (aa 1–554), M-364 (aa 191–554) and C-158 (aa 555–712) (Fig. S6A in Text S1), and examined their abilities to activate IFN-β and NF-κB-specific (PRD-II) promoters in luciferase reporter assays. While N-190 and C-158 were incapable of activating either promoter, overexpression of N-554 and M-364 stimulated both the IFN-β and PRD-II promoters (Fig. S6B in Text S1, left and right, respectively). When co-expressed with wild-type TRIF at a 1∶1 ratio, these fragments did not demonstrate any dominant negative effects (Fig. S6C in Text S1). Other evidence suggests they do not transduce signals from TLR3 (data not shown).

### Altered substrate specificity of 3CD contributes to TRIF cleavage

**Figure 5:**
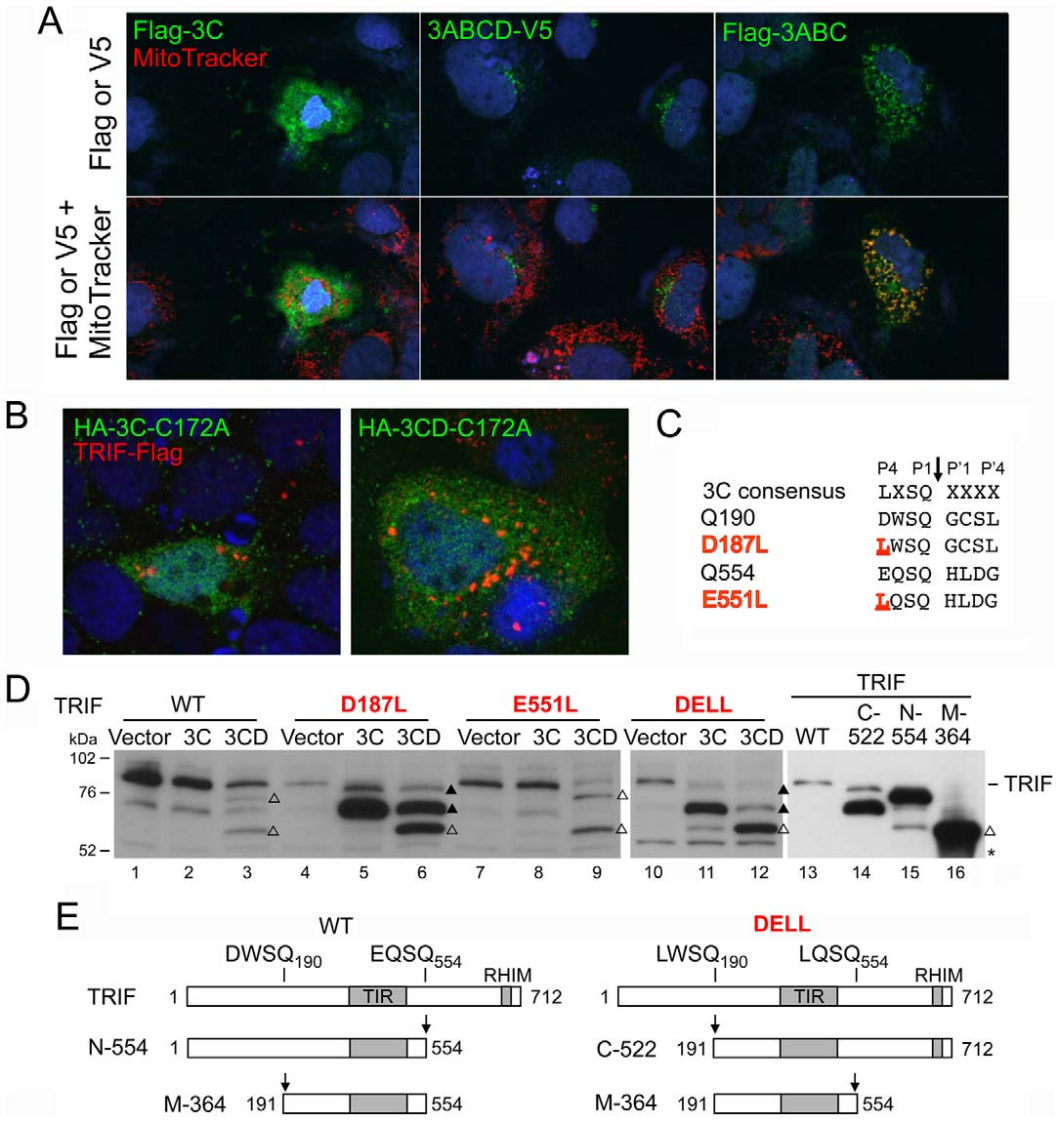
Altered substrate specificity of 3CD contributes to TRIF cleavage. (**A**) Confocal immunofluorescence microscopic images showing subcellular localization of Flag- or V5-tagged 3C^pro^, 3ABCD and 3ABC (green), merged with DAPI (blue) staining of nucleus (top) and additional MitoTracker (red) staining of mitochondria (bottom). (**B**) Confocal immunofluorescence microscopic images of cells expressing C-terminally Flag-tagged full-length TRIF and N-terminally HA-tagged protease-inactive mutant (C172A) forms of 3C^pro^ and 3CD. No co-localization is evident between 3C^pro^ or 3CD proteins and TRIF. (**C**) Alignment of the 3C^pro^ consensus cleavage sequence and TRIF 3CD cleavage sites. The P4 positions at the Q190 and Q554 cleavage sites (D187 and E551, respectively) were substituted with the 3C^pro^-preferred Leu (underlined) in the D187L, E551L and DELL (D187L-E551L) double mutant. (**D**) Cleavage of TRIF mutants by 3C^pro^ and 3CD. HEK 293FT cells were co-transfected with vectors expressing TRIF mutants and 3C^pro^ or 3CD (lane 1 to 12), or transfected with full-length or truncated TRIF constructs corresponding to the cleavage fragments (lane 13–16). Cell extracts were analyzed by immunoblotting of TRIF. The D187L mutant and DELL double mutant were cleaved by both 3C^pro^ and 3CD, resulting in intermediate cleavage fragments (solid triangle) different from that of 3CD-cleaved wild-type (WT) TRIF (open triangle, 75-kDa), but the end product was the same (open triangle, 55-kDa). A nonspecific protein band (‘*’) detected by TRIF antibody indicates equal loading. (**E**) Schematic showing TRIF fragments created by different cleavage order at Q190 vs. Q554, corresponding to cleavage fragments observed in (C) and (D).

We next addressed the question of why TRIF is cleaved by 3CD but very inefficiently or not at all by 3C^pro^. The ability of 3ABC to cleave MAVS, while 3C^pro^ cannot, is related to its unique mitochondrial targeting [12]. To determine if differences in intracellular localization could similarly account for the unique activity of 3CD, we compared the cellular localization of 3C^pro^ and 3ABCD by confocal microscopy. When expressed ectopically with an N-terminal Flag tag, 3C^pro^ was diffusely present throughout the cytoplasm, while Flag-3ABC, included as a control, demonstrated prominent mitochondrial localization, as reported previously [12] (Fig. 5A). In contrast, 3ABCD, expressed with a C-terminal V5 tag, was present at much lower abundance and with a perinuclear, ER-like distribution. Both 3CD and 3ABCD are known to be subject to ubiquitin-mediated proteolysis [28], potentially explaining the low abundance of 3ABCD-V5, much of which is likely processed to 3CD or 3D^pol^. Confocal microscopy of cells ectopically expressing both TRIF and proteolytically-inactive C172A mutants of 3C^pro^ and 3CD revealed no evidence for specific co-localization of these proteins (Fig. 5B). Thus, the unique ability of 3CD to cleave TRIF is not due to its localization to a TRIF-rich compartment.

The noncanonical nature of the 3CD cleavage sites in TRIF, DWSQ_190_ and EQSQ_554_, in which the P4 position is occupied by an amino acid residue with an acidic (Asp or Glu) rather than hydrophobic side chain (Leu, Ile or Val) (Fig. 5C), could explain why TRIF is not efficiently cleaved by 3C^pro^. The fact that they are nonetheless cleaved by 3CD suggests that the substrate specificity of 3CD may differ from that of 3C^pro^ in tolerating or possibly preferring an acidic residue at the P4 position. To assess this potential difference in substrate specificity, we altered the P4 positions within the non-canonical cleavage sites in TRIF, substituting the acidic P4 residues in each with Leu, thereby generating consensus 3C^pro^ sites (TRIF-D187L and TRIF-E551L, respectively, Fig. 5C). When expressed ectopically, the D187L mutant, now carrying a LWSQ cleavage sequence, was readily processed by both 3C^pro^ and 3CD, yielding a novel fragment with an apparent molecular mass of 70 kDa (Fig. 5D, lane 5, 6). This 70 kDa fragment co-migrated in SDS-PAGE with the TRIF C-522 fragment corresponding to aa 191–712 (Fig. 5D, compare lane 5 vs. 14), confirming that cleavage had occurred at Q190. As expected, this fragment was not further cleaved by 3C^pro^, but was further processed by 3CD at Q554, generating the same 55 kDa fragment produced from wild-type TRIF by 3CD (Fig. 5D, lane 3 vs. 6). On the other hand, there was no difference in the processing of the wild-type and the E551L mutant TRIF, the latter of which was only marginally cleaved by 3C^pro^ despite carrying a canonical LQSQ sequence (Fig. 5D, lane 7 vs. 8). Nonetheless, when both cleavage sites were changed to a 3C^pro^ consensus, the resulting double mutant (TRIF-DELL) was readily cleaved by both 3C^pro^ and 3CD (Fig. 5D, lanes 11 and 12). This produced a 70 kDa fragment similar to that observed with the D187L mutant, suggesting that the order of cleavage had been altered to occur first at Q190 (Fig. 5E). Additional processing led to the 55 kDa fragment, although there was less of this product produced by 3C^pro^ than 3CD. Collectively, these results demonstrate that 3CD differs in its substrate specificity from 3C^pro^, tolerating an acidic residue at the P4 position while 3C^pro^ does not, and that this accounts, at least in part, for its ability to cleave TRIF.

### TLR3 signaling and cellular permissiveness to HAV

**Figure 6:**
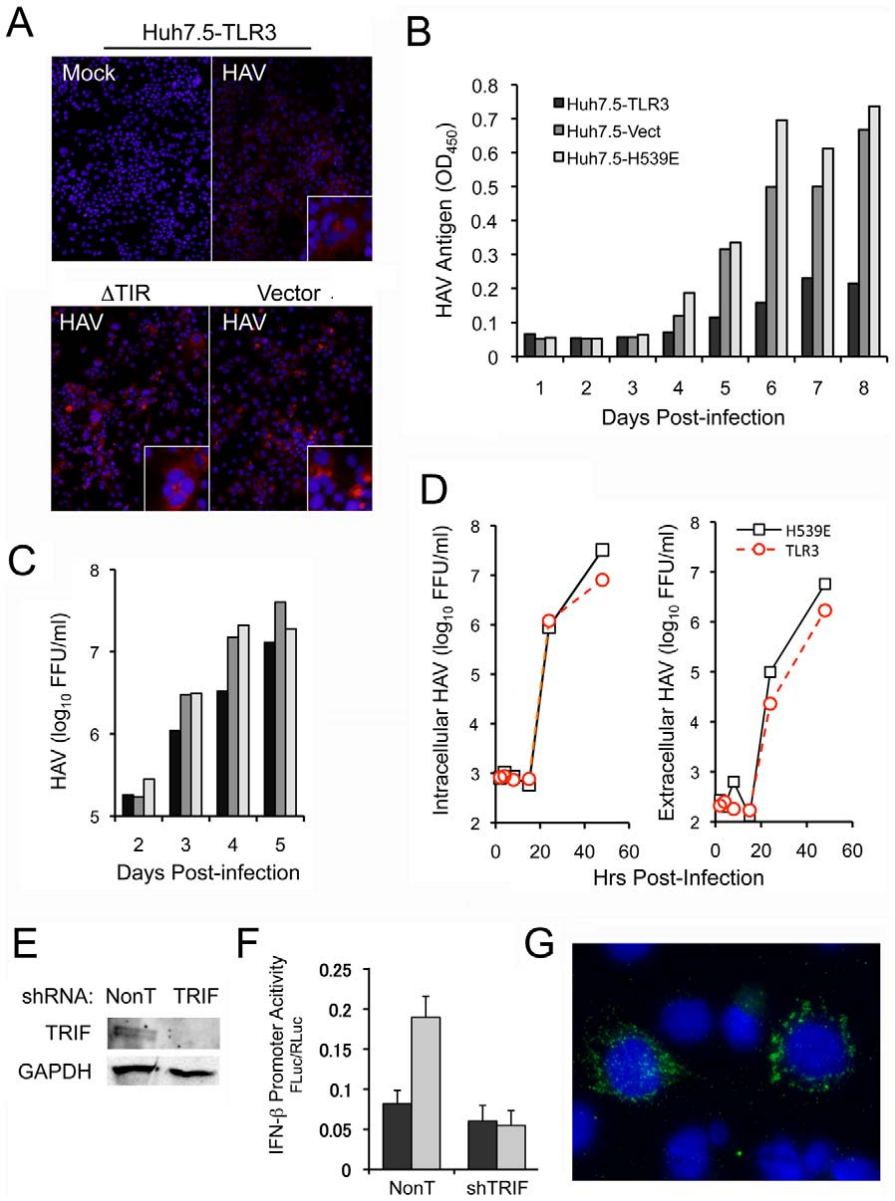
TLR signaling and TRIF regulate cellular permissiveness for HAV. (**A**) Immunofluorescence microscopy of HAV antigen in Huh7.5-TLR3, -ΔTIR, and -Vector cells 5 days after infection of the cells with HAV at an m.o.i. of 1. (**B–C**) Cells were infected with virus at an m.o.i. of 0.05. Virus yields were determined in harvests of the entire culture (cells plus supernatant media) after 3 freeze-thaw cycles. Lysates were clarified by brief centrifugation and supernatants were used for (**B**) HAV antigen ELISA, or (**C**) quantal infectivity assay. (**D**) Paired cultures of Huh7.5-TLR3 and -H539E cells were inoculated with HAV at m.o.i. = 5 to determine one-step replication kinetics. After 1 hr adsorption, cells were washed extensively with PBS, and refed with fresh medium. Culture supernatants (extracellular) and cell lysates (intracellular) were harvested at the times indicated and infectious virus titer determined. (**E**) Immunoblot showing TRIF expression in PH5CH8 cells transduced with lentivirus expressing TRIF-specific shRNA or non-targeting (NonT) shRNA. GAPDH served as a loading control. (**F**) IFN-β promoter activity in cells from panel (E) with (grey bar) and without (black bar) poly-(I:C) stimulation. (**G**) HAV-specific immunofluorescence in PH5CH8 cells after depletion of TRIF. Similar antigen expression was observed very rarely in control cells inoculated in parallel with HAV.

The ability of HAV to antagonize TLR3 signaling is likely to have evolved because antiviral responses evoked by TLR3 act in someway to restrict infection. To test this hypothesis, we assessed viral replication by a variety of methods over a range of multiplicity of infection. Immunoblots demonstrated that viral protein abundance (3C^pro^) was reduced in Huh7.5-TLR3 cells infected at an m.o.i. of 3, compared to cells expressing empty vector or TLR3-ΔTIR (Fig. 1B, lane 4 vs. lanes 2 or 6). Similarly, the fluorescence intensity of HAV antigen was noticeably less in infected Huh7.5-TLR3 cells compared with the control ΔTIR cells (Fig. 6A), although the proportion of cells expressing HAV antigen was not reduced 5 days after infection at an m.o.i. of 1. HAV antigen-specific ELISA assays also showed that Huh7.5-TLR3 cells (infected at an m.o.i. of 0.05) produced less than 50% of the amount of assembled HAV capsid antigen produced by cells expressing empty vector, or a TLR3 mutant incapable of binding dsRNA (TLR3-H539E) [13] (Fig. 6B). Infectious virus yields were also reduced (by 30–60%) in Huh7.5-TLR3 cells infected at low m.o.i. (Fig. 6C).

Somewhat different results were obtained in one-step growth assays done at an m.o.i. of 5.0. HAV replicates very slowly compared to other picornaviruses, with an infectious cycle of approximately 24 hrs evident in such assays (Fig. 6D). Somewhat surprisingly, we observed no differences in the kinetic of intracellular infectious virus accumulation between Huh7.5-TLR3 vs. H539E cells, up to 24 hrs after inoculation of the cells under one-step growth conditions (Fig. 6D, left). Subsequent to this time point, however, less virus was produced in cells expressing functional TLR3. This restriction on virus replication was also reflected in slightly lower (about half log_10_) yields in extracellular infectious virus released from the cells (Fig. 6D). Collectively, these data suggest that TLR3 signaling imposes a modest restriction on HAV infection, particularly at low m.o.i., and after the first round of viral RNA replication. This is reminiscent of the effects of TLR3 expression on low vs. high m.o.i. HCV infections that we have observed in previous studies [13].

To confirm these findings, we sought evidence of a gain in permissiveness for HAV infection in PH5CH8 cells in which TLR3 signaling was impaired by RNAi-mediated depletion of TRIF. PH5CH8 cells are T-antigen transformed adult human hepatocytes that possess robust TLR3 and RLR signaling [14] and are generally nonpermissive for HAV. TRIF was depleted by lentiviral transduction of a TRIF-specific short-hairpin RNA (shRNA) (Fig. 6E), eliminating IFN-β promoter activation by extracellular poly-I:C (Fig. 6F). The cells were infected with HAV at an m.o.i. of 1, and examined 8 days later by immunofluorescence microscopy for viral antigen expression. This was rarely observed in PH5CH8 cells transduced with a non-targeting control shRNA, but detected in ∼2% of the TRIF depleted cells (Fig. 6G). Similar results were obtained in cells transduced with a TLR3-specific shRNA (data not shown).

Thus, reconstitution of TLR3 signaling in Huh7.5 cells results in a modest inhibition of HAV infection, while the ablation of TLR3 signaling in PH5CH8 cells provides a significant replication advantage to HAV. In both cases, these effects are of relatively small magnitude, likely reflecting the presence of redundant innate antiviral defense mechanisms, including responses generated by RLRs or protein kinase R. The abrogation of pro-inflammatory signals, the effects of which cannot be deduced from in vitro experiments, may represent a more substantial advantage to the virus in vivo in HAV-infected persons.

## Discussion

Here, we show that HAV disrupts TLR3 signaling by targeting the essential adaptor protein TRIF for degradation by the 3CD protease-polymerase processing intermediate. The ability of poly-I:C to stimulate the IFN-β promoter or induce the expression of ISGs when added to media was markedly attenuated in HAV-infected Huh7 hepatoma cells in which TLR3 expression had been reconstituted by retroviral gene transduction (Fig. 1A and B). This disruption of TLR3 signaling was associated with a loss of detectable TRIF (Fig. 2), and could be recapitulated by ectopic expression of 3ABCD or 3CD in both Huh7-TLR3 cells and HeLa cells which possess an endogenous TLR3 signaling pathway (Fig. 3). The loss of TRIF expression was linked to the cysteine protease activity residing within the 3C sequence of 3CD, which we demonstrate cleaves TRIF sequentially at two noncanonical 3C^pro^ cleavage sites (Fig. 4B and E). Additional studies suggested that this is due to the ability of the 3D sequence in 3CD to alter the substrate specificity of the protease such that it better accommodates the acidic residues present at the P4 position of cleavage sites in TRIF (Fig. 5D). These observations add to our understanding of the pathogenesis of HAV, a significant human pathogen that has received scant attention in recent years.

**Figure 7:**
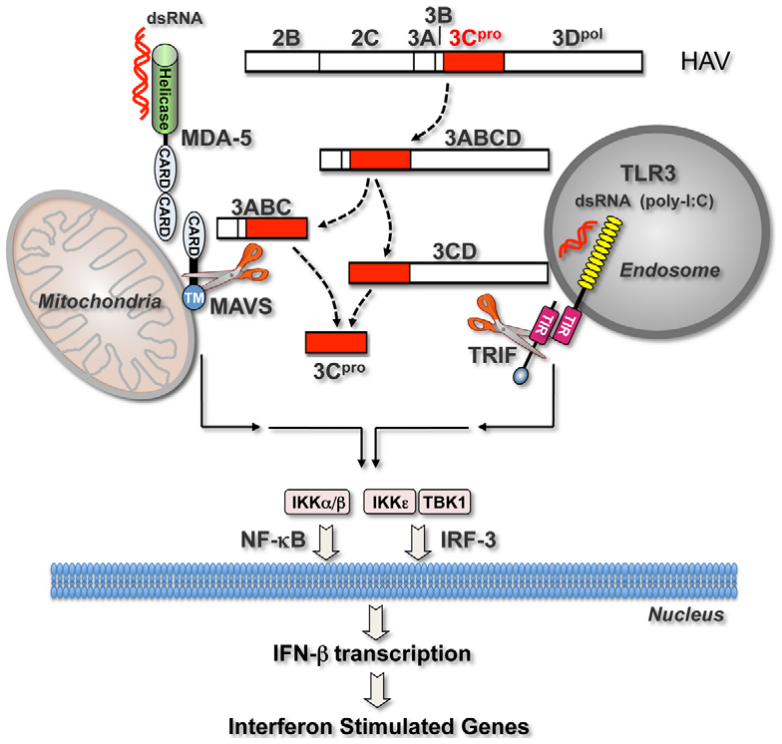
Interferon-activating pathways disrupted during HAV infection by 3C^pro^ precursor-mediated proteolysis of signaling adaptor proteins. Cytosolic HAV RNA is sensed by RNA helicases (most likely MDA-5) which interact through shared caspase-recruitment domains (CARDS) with the adaptor protein, MAVS, localized on the mitochondrial outer membrane. This induces formation of a macromolecular signaling complex that leads to activation of non-canonical (IKKε and TBK-1) and canonical (IKKα/β) kinases of the IκB complex, and subsequent activation of latent cytoplasmic transcription factors, IRF-3 and NF-κB. These activated transcription factors translocate to the nucleus where they induce the transcription of IFN-β mRNA, thereby initiating the production of IFNs and ISGs. TLR3 activates these same transcription factors via a parallel signaling pathway that is initiated upon the sensing of viral dsRNA (or poly-I:C) by TLR3 within an endosomal compartment. Binding of its dsRNA ligand induces the dimerization of TLR3 and subsequent recruitment of the adaptor protein, TRIF, to its cytoplasmic domain through shared Toll/Interleukin-1 receptor (TIR) domains. Additional details of these pathways are available elsewhere [45]. Precursors of the HAV 3C^pro^ cysteine protease block both signaling pathways by directing cleavage of the critical adaptor proteins, MAVS [12] and, as shown in this communication, TRIF. These processing intermediates, 3ABC and 3CD, represent products of alternative processing pathways by which 3C^pro^ is derived from the P2P3 polyprotein fragment (2B to 3D^pol^ shown at the top), a product of the primary HAV polyprotein cleavage between 2A/2B [1]. MAVS cleavage by 3ABC is dependent upon 3ABC localization to the mitochondria, while 3CD cleavage of TRIF, as shown here, is dependent upon altered substrate specificity of 3C^pro^ induced by its fusion to 3D^pol^.

In previous work, we demonstrated that HAV also antagonizes the induction of IFN responses by the cytosolic RLR pattern recognition receptors, RIG-I and MDA-5, by inducing proteolysis of the adaptor protein MAVS [12] (Fig. 7). As we report here with poly-I:C-induced TLR3 signaling, we found that ectopically expressed 3ABCD was capable of disrupting Sendai virus-induced RIG-I signaling. 3ABCD results from secondary processing of the HAV polyprotein at the P2-P3 junction, and is itself subject to further processing via two distinct pathways, one leading to production of 3ABC and the other to 3CD (Fig. 7). Both intermediates contain the catalytically active 3C^pro^ cysteine protease domain, but they have distinct cellular localization and substrate specificities (Fig. 5). 3ABC, due to the presence of a mitochondrial targeting transmembrane domain in 3A, localizes to the mitochondrial membrane where it cleaves MAVS (Fig. 5A). In contrast, 3CD appears to be localized primarily to the perinuclear ER, and its ability to cleave TRIF is dependent upon its unique substrate specificity rather than its intracellular localization (Figs. 5B and 5D).

Our results reveal an unexpected role of the 3D sequence in modulating the substrate specificity of 3CD. 3C^pro^ cleavage sites within the HAV polyprotein, as well as MAVS, contain a hydrophobic amino acid (Leu, Ile, or Val) at the P4 position [12], [24] that fits into the hydrophobic S4 binding pocket within the crystal structure of 3C^pro^ [29],[30]. In contrast, both 3CD cleavage sites within TRIF contain an acidic amino acid residue (Asp-190 and Glu-551) at the P4 position (Fig. 5C), and therefore do not conform to the canonical cleavage sequence. A previous study showed that a peptide substrate with a Glu substitution (underlined) at the P4 position, Ac-EERTQ^↓^SFS-NH2, which is similar to the TRIF cleavage site EQSQ_554_, was not cleaved by 3C^pro^ [31]. Our data suggest that 3CD possesses a unique substrate specificity that allows it to recognize and hydrolyze cleavage sites within TRIF that are otherwise relatively resistant to 3C^pro^. In support of this notion, we showed that changing the non-canonical cleavage site at Gln-190 of TRIF to a canonical 3C^pro^ cleavage sequence resulted in efficient 3C^pro^ proteolysis and a reversal of the order of cleavage at the two sites in TRIF (.5D). A similar change at the Gln-554 site did not make it fully permissive for 3C^pro^ cleavage, however, suggesting that there are other differences in the substrate specificities of 3C^pro^ and 3CD. Our data indicate that the change in substrate specificity of 3CD is conferred *in cis* by the 3D sequence (Fig. 3B), although the structural basis for this remains to be determined.

In addition to their differentiated roles in evading innate immune responses, 3ABC and 3CD are likely to have specialized roles in the viral life cycle. 3ABC is a stable intermediate that is important in processing of the P1-2A segment of the polyprotein required for assembly of the viral capsid [32]. 3CD, based on studies with other picornaviruses, may play a role in the uridylyation of the protein primer of RNA synthesis, 3B (VPg) [33]. The multiple functions of these viral proteins reflect a strategy used by picornaviruses to create processing intermediates that are functionally distinct from their mature products [34], [35], [36].

The dual targeting of RLR and TLR3 signaling by HAV 3ABCD processing intermediates is reminiscent of the HCV NS3/4A protease, which disrupts both RIG-I and TLR3 signaling pathways by proteolytically cleaving the same signaling adaptor proteins, MAVS and TRIF, respectively [7], [8], [9]. 3C^pro^ and NS3/4A are both chymotrypsin-like proteases with double β-barrel folds [30], [37], but they are not closely related phylogenetically. The HAV 3C^pro^ protease has a cysteine nucleophile in its active site, while NS3/4A has a serine. These viral proteases have very different substrate specificities, and they cleave MAVS and TRIF at distinctly different sites [7,8,9,12, and Fig. 3]. The fact that both of these hepatotropic viruses express proteases targeting these two critical adaptor molecules is thus a remarkable example of convergent evolution. It also speaks strongly to the importance of these signaling pathways in the control of RNA viruses in the liver. However, since HAV infection is always successfully controlled by the host (except in rare cases of fulminant disease), these data indicate that the disruption of RLR and TLR3-mediated antiviral defenses is not sufficient for a virus to establish the longterm persistence that typifies most HCV infections. HCV must possess additional immune evasion strategies to account for its unique capacity to establish chronic infections.

We demonstrated a minimal gain of permissiveness for HAV replication in hepatocyte-derived cells in which TLR3 or TRIF expression was depleted (Fig. 6G), and a reduction in viral antigen expression in hepatoma cells with active TLR3 signaling (Fig. 6B). However, these effects were modest, potentially reflecting very efficient control of TLR3 signaling by 3CD in infected cells such that TLR3 has little impact on viral replication. Alternatively, it may be that the primary advantage gained by HAV in antagonizing TLR3 signaling is impaired production of proinflammatory cytokines and reduced inflammation associated with the infection. TLR3 signaling is critically important to murine host defense against coxsackievirus B, another picornavirus [38], and it is plausible that the disruption of TLR3 signaling has significance beyond impairing the type I IFN response.

The subversion of both RLR and TLR3 signaling likely contributes to the relatively lengthy, clinically silent incubation period that precedes acute liver injury in hepatitis A. This period is characterized by robust viral replication within the liver and shedding of virus in feces, which reaches a maximum at the onset of hepatic inflammation [10], [39]. The absence of a type I IFN response in acute infectious hepatitis was hinted at in clinical studies done almost 40 years ago [40]. Consistent with this, we recently documented a paucity of type I IFN-dependent ISG expression (*e.g.*, IFIT-1, ISG15) within the liver of HAV-infected chimpanzees during the first weeks of infection despite high viral RNA copy numbers [41]. The cleavage of MAVS and TRIF by 3ABC and 3CD, respectively, provides a partial mechanistic explanation for this. By dealing a double blow to two major cellular antiviral response pathways, HAV appears able to block somatic cell expression of IFN-α/β, thus facilitating its replication. Yet to be explained is how it evades recognition by plasmacytoid dendritic cells (pDCs), which may play a significant role in sensing HCV infection in the liver and generating the strong intrahepatic ISG responses that are often observed in acute and chronic hepatitis C [42], [43].

## Methods

### Cells and viruses

HEK 293FT cells, Huh7, Huh-7.5 and Bla-C cells [12] were cultured in DMEM with 8% FBS. Huh7-TLR3, Huh-7.5-TLR3 and related control cells [13], and HAV-Bla subgenomic replicon cells were cultured in the same medium supplemented with blasticidin [12]. The cell culture-adapted HAV strain HM175/18f [16] was amplified in Huh7 cells; on fetal rhesus kidney FRhK4 cells.

### Plasmids and antibodies

pCDNA6-TRIF [7] and pCMV-HA vectors expressing N-terminally HA-tagged HAV proteins derived from HM-175/18f virus [12] have been described previously. Similar pCMV-HA vectors expressing the wild-type HM175 3C^pro^ and 3CD proteins were constructed by amplification of the corresponding sequences from pHAV/8y (Suzanne Emerson, NIAID). Truncations of TRIF were generated by PCR mutagenesis, and mutations in TRIF and 3CD constructed by site-directed mutagenesis (Strategene). Other plasmids were obtained from the following sources: pIFN-β-Luc (Rongtuan Lin, McGill University), pPRD-II-Luc (Michael Gale, University of Washington), pCMV-β-gal (Clontech), pRL-CMV (Promega), pEF-Bos-TRIF (Kate Fitzgerald, University of Massachusetts) and pCDNA3-Flag-IKKε (Tom Maniatis, Harvard University). Antibodies used in these studies included: anti-TLR3 (Ilkka Julkunen, National Institute for Health and Welfare, Finland), anti-TRIF S219 (Cell Signaling Technology), anti-TRIF aa 4–31 (Alexia), anti-IRF-3 sc-9082 (Santa Cruz), monoclonal anti-HAV K2-4F2, K3-4C8 (Commonwealth Serum Laboratories, Victoria, Australia), and 6A5 (John Hughes, Merck, Sharp & Dohme), anti-HAV 2A (David Sangar, Wellcome Biotech), anti-3C^pro^ (Verena Gauss-Muller, University of Lübeck), anti-ISG15 (Santa Cruz), and anti-HA and anti-Actin (Sigma). Rabbit anti-TRIF antibody S537-2 was obtained by immunization of rabbits with recombinant TRIF protein [7].

### Transfection and luciferase reporter assays

For protein expression, cells were transfected with plasmid DNA using Lipofectamine 2000 (Invitrogen) and lysates prepared 20 hrs later using 1% NP-40 lysis buffer. HAV-infected cells (m.o.i. = 3) were cultured for 4 days prior to transfection. For luciferase reporter assays, expression and/or Luc reporter plasmids were transfected into cells (seeded in triplicate in 96-well format) with an internal β-galactosidase (pCMV-β-gal) or Renilla luciferase (pRL-CMV, Promega) transfection control. At 20 hours posttransfection, when indicated, poly(I:C) (Sigma) was added to the medium and cells incubated for additional 6 hours. Cells were lysed in Reporter Lysis Buffer (Promega) and equal quantity of lysate used for luciferase and β-galactosidase assays (Promega). In experiments using the Renilla luciferase control, cells were lysed in Passive Lysis Buffer (Promega) and tested by Dual-Luciferase assay (Promega). For each sample, the luciferase activity was normalized to the β-galactosidase or renilla luciferase activity. In the case of poly(I:C) stimulation, results were presented as fold induction compared to unstimulated cells. Statistical analysis was performed using two-tailed Student's t test.

### HAV assays

HAV antigen-specific ELISA was carried out using a post-convalescent human antibody for capture [44], and a murine monoclonal antibody (K24F2) for detection. Absorption at 450 nm was determined on a Synergy (Biotek, Inc) plate reader. The infrared fluorescent immunofocus assay (IR-FIFA) for infectious HAV was done using FRhK-4 cells as previously described [12]. For detection of HAV antigen by immunofluorescence microscopy, cells were fixed with 4% PFA for 25 min, labeled with murine mAb 6A5 and after extensive washing incubated with goat anti-mouse IgG Alexa-594 conjugate (Invitrogen).

### Expression and purification of GST-3C from *E. coli*

The 3C^pro^ coding sequence from HAV strain HM175/18f was cloned into bacterial expression vector pGEX-4T3 (GE Life Sciences), fused in-frame with an N-terminal GST tag. For protein expression, an overnight culture of *E. coli* strain BL21(DE) (Novagen) containing the expression construct was diluted 10-fold and cultured at 37°C for 2 hrs. Expression was induced by addition of 0.1 mM IPTG and continued culture at 25°C for 3 hrs. Bacterial cells were harvested and lysed in BugBuster solution (EMD Biosciences) containing 37.5 U/ml Benzonase, 15 KU/ml recombinant lysozyme and 2 mM DTT. GST-3C^pro^ fusion protein was purified from the bacterial lysate by affinity chromatography using the GST MicroSpin Purification Module (GE Life Sciences) according to the manufacturer's instructions.

### *In vitro* cleavage assay

Myc-TRIF and its truncated forms were synthesized *in vitro* and labeled with [^35^S]-Met/Cys using T7 Coupled Transcription/Translation System (TNT, Promega) according to the manufacturer's instructions. Cleavage assays were performed in a 10-µl mixture containing 1 µl TNT product and 0.5 µM purified GST-3C^pro^ in a buffer containing 50 mM Tris-HCl pH 8.0, 2.5 mM EDTA and 2 mM DTT. Similarly purified GST was used as a negative control at the same concentration. Reactions were carried out overnight at 3°C and stopped by addition of an equal volume of 2X SDS sample buffer. Cleavage products were analyzed by SDS-PAGE followed by autoradiography.

### Immunoprecipitation and immunoblotting

For detection of endogenous TRIF, 1 mg of cell lysate was immunoprecipitated with 1 µg of rabbit anti-TRIF antibody S537-2 [7], followed by immunoblotting with anti-TRIF S219. HA-3CD was detected with a similar method using anti-HA for immunoprecipitation and anti-3C^pro^ for immunoblotting.

### IRF-3 dimerization

Huh7-TLR3 cells were mock-infected or infected with HAV at m.o.i. = 5 and cultured for 5 days, then stimulated by the addition of poly-(I:C) (50 µg/ml) to the medium for 2 hours and lysed with 1% NP-40 lysis buffer. Cell lysates (10 µg) were mixed with deoxycholate (DOC) sample buffer (final concentration 1% DOC) and separated by Tris-Glycine/1% DOC native PAGE. IRF-3 monomer and dimer were detected by immunoblotting with rabbit anti-IRF-3 sc-9082.

### Laser-scanning confocal microscopy

Ectopically expressed Flag-tagged TRIF, 3C^pro^ and 3ABC, HA-tagged 3C^pro^ and 3CD, and V5-tagged 3ABCD were imaged as described previously [12]. For visualization of poly(I:C)-induced changes in IRF-3 localization, Huh7-TLR3 cells were grown on chamber slides, infected at low m.o.i. and cultured for 5 days. Cells were then mock-stimulated or stimulated with poly-(I:C) (50 µg/ml) added to the medium for 2 hours, and fixed with 4% paraformaldehyde. After permeabilization with 0.25% Triton X-100, the cell monolayer was incubated with rabbit anti-IRF-3 sc-9082 and murine anti-HAV K3-4C8, followed by secondary antibodies goat anti-rabbit Alexa Fluor 488 and goat anti-mouse Alexa Fluor 594 (Invitrogen). Nuclei were counterstained stained with DAPI. Images were collected using a Leica DMIRB Inverted Microscope in the Michael Hooker Microscopy Facility.

### GenBank accession numbers

HAV strain HM175/18f (including individual HAV proteins), M59808; wild-type HAV strain HM175, M14707.1; TLR3, NP_003256; ISG15, NP_005092; TRIF (TICAM-1), NP_891549; IKKe, NP_054721; IRF-3, NP_001562; MDA5, NP_071451; RIG-I, O95786; MAVS (IPS-1, Cardif, VISA), Q7Z434; GAPDH, P04406; Actin, P60709.

## Supporting Information

Text S1 Supporting figures and legends.
(PDF)
