# Supplementary material for: Disruption of TLR3 Signaling Due to Cleavage of TRIF by the Hepatitis A Virus Protease-Polymerase Processing Intermediate, 3CD
Source: PLoS Pathog. 2011 Sep 8;7(9):e1002169. doi: 10.1371/journal.ppat.1002169 (PMC3169542; doi:10.1371/journal.ppat.1002169)
Supplement: Text S1 — Supporting figures and legends. (PDF) [file ppat.1002169.s001.pdf]

# SUPPORTING INFORMATION

# Disruption of TLR3 signaling due to cleavage of TRIF by the hepatitis A virus protease-polymerase processing intermediate, 3CD

Lin Qu, Zongdi Feng, Daisuke Yamane, Yuqiong Liang,
Robert E. Lanford, Kui Li, and Stanley M. Lemon

## SUPPORTING FIGURES

### Figure S1

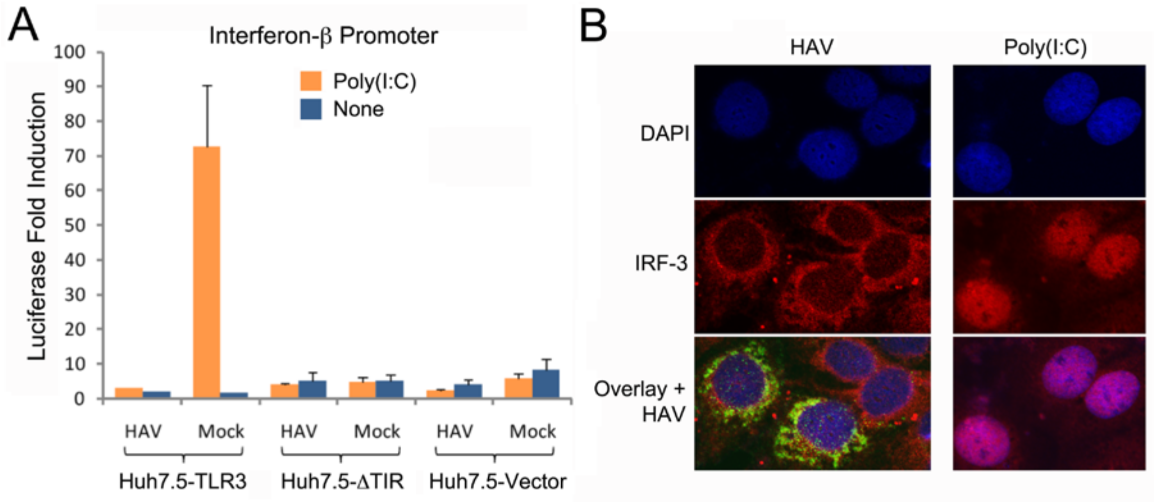


**Figure S1. HAV inhibits TLR3 signaling in Huh7.5-TLR3 cells that stably express functional TLR3. (A)** Huh7.5-TLR3 cells, Huh7.5-ΔTIR or Huh7.5-Vect cells (Huh-7.5 cells transduced with an empty vector) [1], were mock- or HAV-infected (m.o.i. = 3) for 4 days, transfected with an IFN-β-Luc reporter plasmid, and stimulated with extracellular poly(I:C) for 6 hours. Luciferase activity is presented as fold induction by poly(I:C). **(B)** Laser-scanning confocal microscopy showing that HAV infection (48 hrs) of Huh7-TLR3 cells does not result in activation of the transcription factor IRF-3 (red), as inferred from the lack of nuclear translocation. (left panels) Cells were infected with HAV at low m.o.i., without poly(I:C) stimulation. Infected cells are identified by labeling (green) with K34C8 monoclonal antibody. IRF-3 is localized to the cytoplasm in both infected and uninfected cells. (right panels) Similar imaging of uninfected cells. IRF-3 is strongly partitioned to the nucleus 2 hrs following the addition of poly(I:C) (50 μg/ml) to media. Cells were counterstained with DAPI (blue) to visualize nuclei.

**Figure S2.**

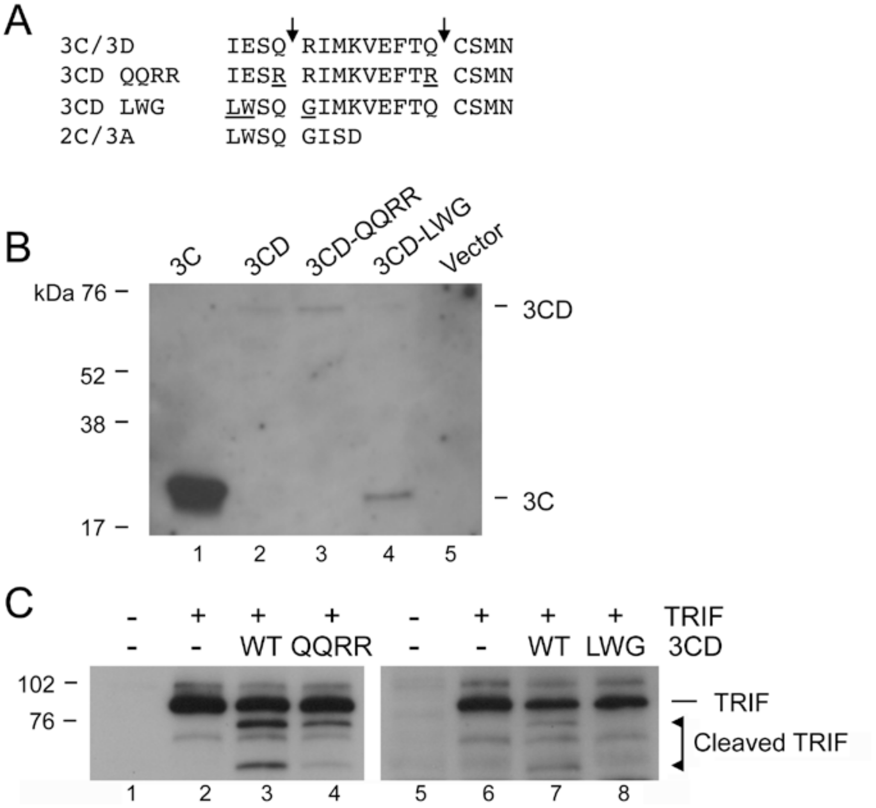


**Figure S2**. **Cleavage of TRIF is modulated by 3CD mutations that alter the efficiency of *cis*-cleavage at the 3CD junction**. **(A)** Alignment of wild-type and mutated 3C/3D cleavage sequences, showing as a reference the sequence at the HAV 2C/3A junction. Arrows indicate sites of scission. Residues altered in the mutants are underlined and either eliminate (QQRR) or optimize (LWG) the 3C/3D cleavage. In the LWG mutant, the P4-P2' residues of the modified 5' 3C/3D principal cleavage site, LWGQ↓GI, are identical to those at the 2C/3A junction. **(B)** Immunoblot showing that the mutations at the 3C/3D cleavage sites result in hypo- or hyper-processing of 3CD. HEK 293FT cells were transfected with HA-tagged 3C (control) or mutated 3CD constructs. Cell lysates were analyzed by anti-HA immunoprecipitation followed by immunoblotting with antibody to $3C^{pro}$. **(C)** Cleavage of TRIF by 3CD hypo- or hyper-processing mutants. HEK 293FT cells were co-transfected with vectors expressing TRIF and 3CD mutant constructs. TRIF and TRIF cleavage products (marked by arrows) were detected by immunoblotting.

**Figure S3.**

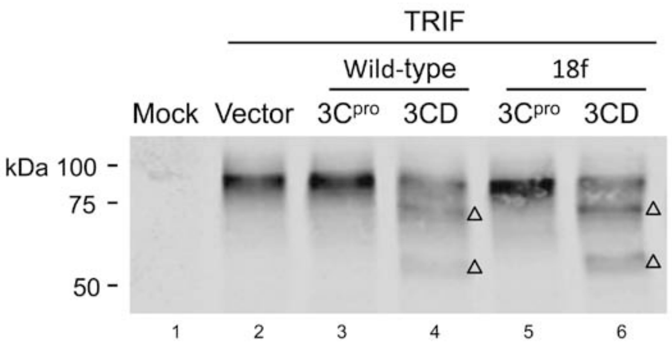


**Figure S3. Cleavage of TRIF by wt HM175 strain HAV 3CD.** HM175/18f virus contains numerous cell culture-adaptation mutations, including amino acid substitutions in both 3C$^{pro}$ and 3CD [4]. To confirm that these mutations are not responsible for the TRIF cleavage phenotype of the HM175/18f 3CD processing intermediate, 3C$^{pro}$ and 3CD sequences from the wt HM175 (lanes 3 and 4) and HM175/18f (lanes 5 and 6) viruses were co-expressed as N-terminally HA-tagged molecules with ectopic TRIF (lanes 2-6) in 293FT cells. Immunoblots for TRIF were subsequently carried out on protein extracts. Expression of 3CD from both wt HM175 and HM175/18f resulted in TRIF degradation (△ = major cleavage product), while this was not observed with 3C$^{pro}$ from either HM175 variant.

**Figure S4**

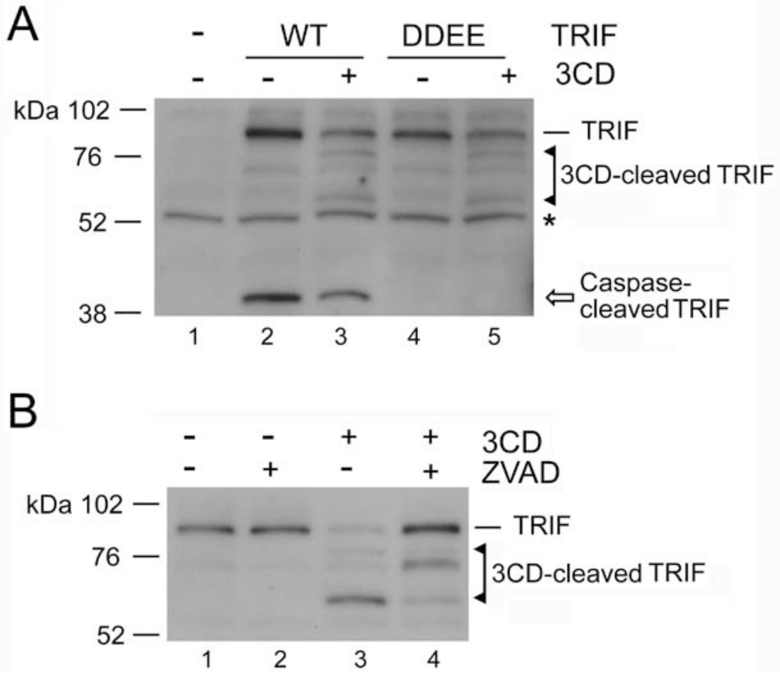


**Figure S4. A caspase-resistant TRIF mutant remains susceptible to 3CD cleavage, while 3CD cleavage of TRIF but is partially blocked by the caspase inhibitor, z-VAD-fmk**. **(A)** 3CD-mediates cleavage of both wild-type (WT) and a caspase-resistant D281E-D289E (DDEE) TRIF double mutant [2]. HEK 293FT cells were co-transfected with vectors expressing wt and mutant TRIF and 3CD. TRIF and cleavage products generated by 3CD or caspases were analyzed by immunoblotting. A nonspecific protein band (marked by asterisk) detected by TRIF antibody indicates equal loading. **(B)** z-VAD-fmk modulates 3CD cleavage of TRIF. HEK 293FT cells were co-transfected with TRIF and 3CD expression vectors. At 4 hours posttransfection, z-VAD-fmk was added to the medium and incubated for an additional 16 hours. TRIF and related cleavage products were analyzed by immunoblotting.

**Figure S5.**

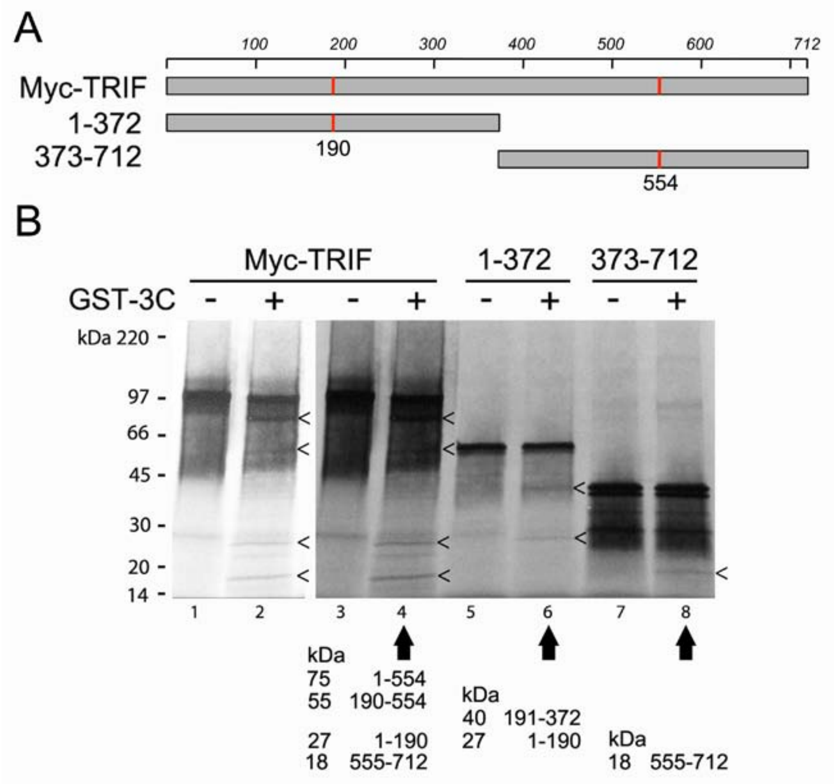


**Figure S5. Cleavage of *in vitro*-translated TRIF by purified GST-3C$^{pro}$. (A)** Schematics of Myc-tagged full-length and truncated forms of TRIF, showing 3CD cleavage sites Q190 and Q554. **(B)** Cleavage of *in vitro*-translated Myc-TRIF by purified bacterially-expressed GST-3C$^{pro}$. *In vitro*-translated, [$^{35}$S]-labeled full-length (aa 1-712) and truncated (aa 1-372 and 373-712) forms of Myc-TRIF were incubated with GST-3C$^{pro}$ (at a final concentration of 0.5 μM) purified from *E. coli*. TRIF cleavage fragments are marked by arrow heads, and the identity of each shown below lanes 4, 6 and 8. Some fragments (such as the 40-kDa fragment in lane 6) migrates with an aberrantly high apparent molecular mass due to their proline-rich nature [3]. Lanes 1-2 are identical to lanes 3-4, but with the gamma adjusted to better visualize the 75- and 55-kDa cleavage fragments.

**Figure S6.**

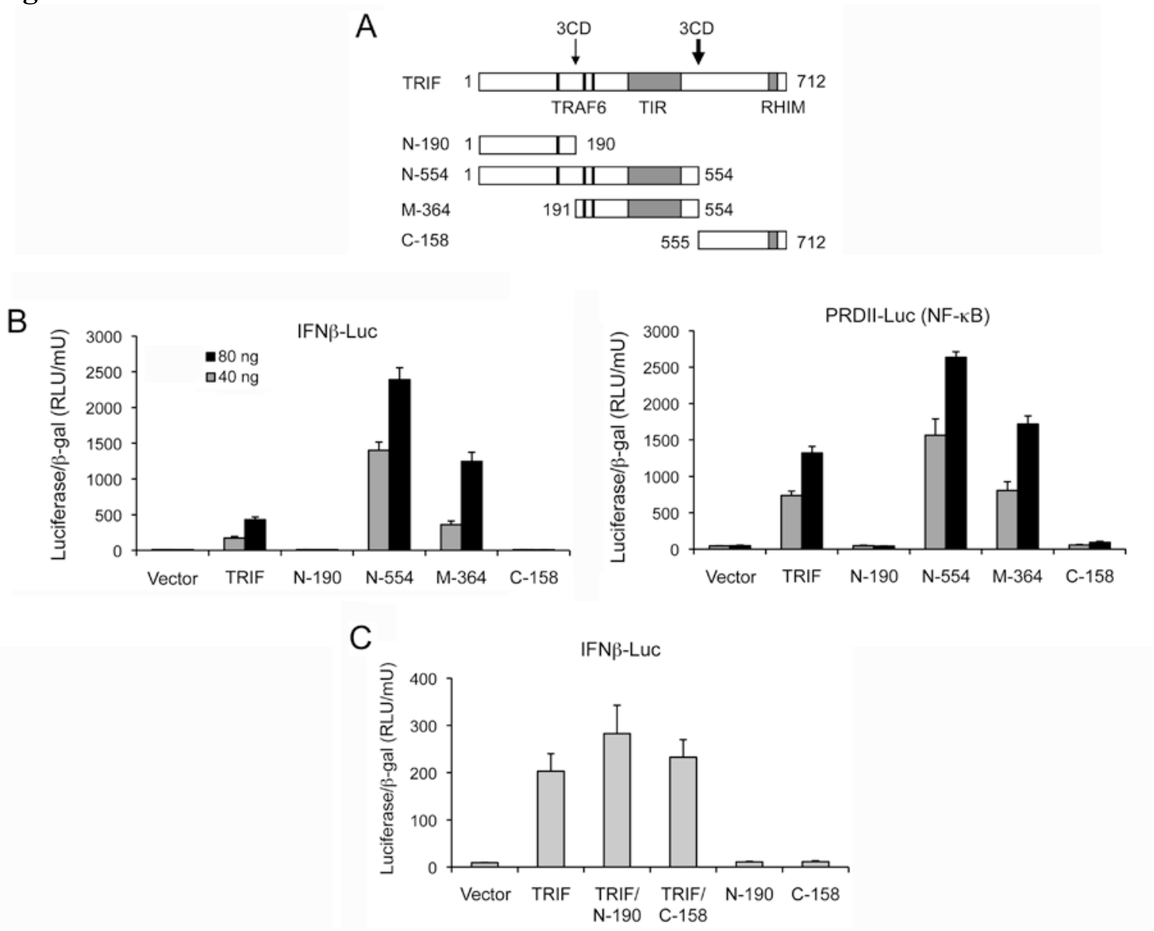


**Figure S6**. **Signaling activation by overexpression of 3CD-cleavage fragments of TRIF**. **(A)** Schematics of TRIF fragments resulting from 3CD cleavage, showing TRAF6 binding motifs (bars) and TIR and RHIM domains (gray boxes) in individual fragments. **(B)** Dose-dependent activation of IFN-β-Luc promoter (left) and NF-κB-specific PRDII-Luc promoter (right) following ectopic expression of TRIF fragments in HEK 293FT cells. **(C)** TRIF-induced activation of IFN-β-Luc promoter in HEK 293FT cells was not affected by co-expression (at a 1:1 ratio) with the N-190 or C-158 fragment. In (B) and (C), luciferase reporter activity was normalized to an internal β-gal transfection control.

## SUPPORTING REFERENCES

1. Wang N, Liang Y, Devaraj S, Wang J, Lemon SM, et al. (2009) Toll-like receptor 3 mediates establishment of an antiviral state against hepatitis C virus in hepatoma cells. J Virol 83: 9824-9834.
2. Rebsamen M, Meylan E, Curran J, Tschopp J (2008) The antiviral adaptor proteins Cardif and Trif are processed and inactivated by caspases. Cell Death Differ 15: 1804-1811.
3. Ferreon JC, Ferreon ACM, Li K, Lemon SM (2005) Molecular determinants involved in TRIF proteolysis by the hepatitis C virus NS3/4A protease J BiolChem 280: 20483-20492.
4. Lemon SM, Murphy PC, Shields PA, Ping LH, Feinstone SM, et al. (1991) Antigenic and genetic variation in cytopathic hepatitis A virus variants arising during persistent infection: evidence for genetic recombination. Journal of Virology 65: 2056-2065.
